# Final Results from a Phase II Trial of Osimertinib for Elderly Patients with Epidermal Growth Factor Receptor t790m-Positive Non-Small Cell Lung Cancer That Progressed during Previous Treatment

**DOI:** 10.3390/jcm9061762

**Published:** 2020-06-05

**Authors:** Akira Nakao, Osamu Hiranuma, Junji Uchino, Chikara Sakaguchi, Tomoyuki Araya, Noriya Hiraoka, Tamotsu Ishizuka, Takayuki Takeda, Masayuki Kawasaki, Yasuhiro Goto, Hisao Imai, Noboru Hattori, Keita Nakatomi, Hidetaka Uramoto, Kiyoaki Uryu, Minoru Fukuda, Yasuki Uchida, Toshihide Yokoyama, Masaya Akai, Tadashi Mio, Seiji Nagashima, Yusuke Chihara, Nobuyo Tamiya, Yoshiko Kaneko, Takako Mouri, Tadaaki Yamada, Kenichi Yoshimura, Masaki Fujita, Koichi Takayama

**Affiliations:** 1 Department of Respiratory Medicine, Faculty of Medicine, Fukuoka University, Fukuoka 814-0180, Japan; akiran@fukuoka-u.ac.jp (A.N.); mfujita@fukuoka-u.ac.jp (M.F.); 2 Department of Respiratory Medicine, Otsu City Hospital, Shiga 520-0804, Japan; osamu319@true.ocn.ne.jp; 3 Department of Pulmonary Medicine, Kyoto Prefectural University of Medicine, Kyoto 602-8566, Japan; c1981311@koto.kpu-m.ac.jp (Y.C.); koma@koto.kpu-m.ac.jp (N.T.); kaneko-y@koto.kpu-m.ac.jp (Y.K.); tmouri@koto.kpu-m.ac.jp (T.M.); tayamada@koto.kpu-m.ac.jp (T.Y.); takayama@koto.kpu-m.ac.jp (K.T.); 4 Department of Pulmonary Medicine, Rakuwakai Otowa Hospital, Kyoto 607-8062, Japan; rakuwadr1141@rakuwadr.com; 5 Department of Respiratory Medicine, National Hospital Organization, Kanazawa Medical Center, Ishikawa 920-8650, Japan; komatsu_alone@yahoo.co.jp; 6 Department of Respiratory Medicine, Japanese Red Cross Kyoto Daiichi Hospital, Kyoto 605-0981, Japan; noriya-hiraoka@kyoto1-jrc.org; 7 Third Department of Internal Medicine, Faculty of Medical Sciences, University of Fukui, Fukui 910-1193, Japan; tamotsui@u-fukui.ac.jp; 8 Department of Respiratory Medicine, Japanese Red Cross Society Kyoto Daini Hospital, Kyoto 602-8026, Japan; dyckw344@yahoo.co.jp; 9 Department of Respiratory Medicine, National Hospital Organization, Omuta National Hospital, Fukuoka 837-0911, Japan; kawasaki.masayuki.hx@mail.hosp.go.jp; 10 Department of Respiratory Medicine, Fujita Health University, Aichi 470-1192, Japan; gotoyasu@fujita-hu.ac.jp; 11 Division of Respiratory Medicine, Gunma Prefectural Cancer Center, Gunma 373-8550, Japan; hi-imai@gunma-cc.jp; 12 Department of Molecular and Internal Medicine, Graduate School of Biomedical & Health Sciences, Hiroshima University, Hiroshima 734-8553, Japan; nhattori@hiroshima-u.ac.jp; 13 Department of Respiratory Medicine, Kyushu Central Hospital of the Mutual Aid Association of Public School Teachers, Fukuoka 815-8588, Japan; keita.nakatomi@icloud.com; 14 Department of Thoracic Surgery, Kanazawa Medical University, Ishikawa 920-0293, Japan; hidetaka@kanazawa-med.ac.jp; 15 Department of Respiratory Medicine, Yao Tokushukai General Hospital, Osaka 581-0011, Japan; kiyoaki.uryuu@tokushukai.jp; 16 Second Department of Internal Medicine, Nagasaki University Hospital, Nagasaki 852-8523, Japan; mifukuda@nagasaki-u.ac.jp; 17 Department of Respiratory Medicine, Shiga University of Medical Science Hospital, Shiga 520-2192, Japan; uchiy@belle.shiga-med.ac.jp; 18 Department of Respiratory Medicine, Kurashiki Central Hospital, Okayama 710-8602, Japan; ty14401@kchnet.or.jp; 19 Department of Respiratory Medicine, Japanese Red Cross Fukui Hospital, Fukui 918-8501, Japan; makai-0314@fukui-med.jrc.or.jp; 20 Division of Respiratory Medicine, Center for Respiratory Diseases, National Hospital Organization Kyoto Medical Center, Kyoto 612-8555, Japan; tmio.kmc@gmail.com; 21 Department of Respiratory Medicine, National Hospital Organization Nagasaki Medical Center, Nagasaki 856-0835, Japan; nagashima.seiji.kg@mail.hosp.go.jp; 22 Center for Integrated Medical Research, Hiroshima University Hospital, Hiroshima University, Hiroshima 734-8553, Japan; keyoshim@hiroshima-u.ac.jp

**Keywords:** non-small cell lung cancer, EGFR-TKI, T790M, osimertinib

## Abstract

Epidermal growth factor receptor tyrosine kinase inhibitors (EGFR-TKIs) are used for treating EGFR-mutated lung cancer, and osimertinib is effective in cases that acquired T790M mutations after treatment with the first- and second-generation EGFR-TKIs. However, no study has evaluated its safety and efficacy in older patients. This phase II trial (jRCTs071180002) evaluated osimertinib in T790M mutation-positive Japanese patients who were ≥75 years old and had experienced relapse or progression after previous EGFR-TKI treatment. Our previous report that enrolled 36 patients showed the overall response rate (58.3%) and disease control rate (97.2%), while this report describes the results for the progression-free survival (PFS), overall survival (OS), and safety analyses. The median PFS was 11.9 months (95% confidence interval (CI): 7.9–17.5), and the median OS was 22.0 months (95% CI: 16.0 months–not reached). The most frequent adverse events were anemia/hypoalbuminemia (27 patients, 75.0%), thrombocytopenia (21 patients, 58.3%), and paronychia/anorexia/diarrhea/neutropenia (15 patients, 41.7%). Pneumonitis was observed in four patients (11.1%), including two patients (5.6%) with Grade 3–4 pneumonitis. These results suggest that osimertinib was relatively safe and effective for non-small cell lung cancer that acquired T790M mutations after previous EGFR-TKI treatment, even among patients who were ≥75 years old.

## 1. Introduction

Treatment for epidermal growth factor receptor (EGFR)-mutated non-small cell lung cancer (NSCLC) typically involves EGFR tyrosine kinase inhibitors (EGFR-TKIs). Gefitinib and erlotinib are the first-generation EGFR-TKIs that provide significant survival benefits compared with platinum-based chemotherapy in clinical trials [1,2,3,4,5,6]. Afatinib and dacomitinib are the second-generation EGFR-TKIs that provide significantly longer progression-free survival (PFS) compared to that of platinum-based chemotherapy and first-generation EGFR-TKIs, although the second-generation EGFR-TKIs did not significantly improve overall survival (OS) [7,8,9,10,11]. In addition, these drugs are associated with more severe toxicity profiles, such as skin disorders, relative to the first-generation EGFR-TKIs.

Various mechanisms are responsible for resistance to the first-generation and second-generation EGFR-TKIs, with more than one-half of the cases involving the EGFR exon 20 T790M mutation [12]. Osimertinib is a third-generation EGFR-TKI that was developed to address this issue [12], and the AURA3 study revealed that it provided significantly longer PFS compared to platinum-based chemotherapy among patients with T790M-mutated lung cancer [13]. Moreover, the FLAURA trial conducted on first-line treatment revealed that osimertinib administered as an initial treatment for EGFR-mutated cases significantly prolonged PFS and OS compared with the first-generation EGFR-TKIs, with a median OS of >3 years [14,15]. Furthermore, osimertinib is expected to have good central nervous system translocation and a limited inhibition of the wild-type EGFR, which may make it less toxic, and therefore, the first choice for EGFR-mutated NSCLC [16,17,18]. Nevertheless, additional evidence is needed to support this application based on various patient populations. We have performed a phase II study to investigate the efficacy and safety of osimertinib in elderly Japanese patients (≥75 years old) with NSCLC containing the T790M mutation who progressed or experienced a relapse while receiving the first- and second- generations of EGFR-TKI treatment. In our previous report, the response rate was the primary endpoint, and the disease control rate was the secondary endpoint [19]. This report presents the results from our final analyses of PFS, OS, and safety events, which were the additional secondary endpoints in that trial.

## 2. Experimental Section

### 2.1. Patients

The study eligibility and exclusion criteria have been previously reported [19,20]. Patients were enrolled in this study between July 2016 and May 2018 if they met the following eligibility criteria: recurrence of NSCLC after achieving stable disease or better as their best overall response after treatment with the first- and second-generation of EGFR-TKIs; harboring an EGFR mutation (activating) and being T790M-positive; aged over 75 years; performance status of ≤1 based on the Eastern Cooperative Oncology Group (ECOG) scale; adequate bone marrow function (leukocyte count 3000–12,000/µL, platelet count ≥100,000/µL, and hemoglobin level ≥9.0 g/dL), adequate hepatic function (bilirubin level ≤1.5 mg/dL, aspartate aminotransferase of ≤100 IU/L, alanine aminotransferase of ≤100 IU/L), and adequate renal function (serum creatinine ≤2.0 mg/dL); a measurable lesion according to the Response Evaluation Criteria in Solid Tumors (RECIST) guidelines version 1.1; and provision of written informed consent. The exclusion criteria were pulmonary disorders; including idiopathic pulmonary fibrosis; interstitial pneumonia; pneumoconiosis; active radiation pneumonitis and drug-induced pneumonia, active infection; symptomatic brain metastasis; uncontrollable diabetes mellitus or severe comorbidities such as heart disease or renal disease; watery diarrhea; active concomitant malignancy; pregnancy or other medical problems that could prevent compliance with the protocol. The trial protocol was registered at Japan Registry of Clinical Trials (jRCTs071180002) and was approved by the ethical review board of Clinical Research Network Fukuoka Certified Review Board (CRB7180004). All patients provided written informed consent before enrollment.

### 2.2. Study Design, Treatments, and Endpoints

This single-arm-multicenter study involved daily oral administration of osimertinib (80 mg/day). Osimertinib had to be started at 80 mg/day, and if adverse events (AEs) occurred, dose reduction was performed according to the dose reduction criteria. Administration of osimertinib was continued until the patient met the discontinuation criteria or disease progression. Tumor assessments were performed at baseline, every 6 weeks (± 2 weeks) for 6 months, and then every 9 weeks (± 2 weeks) until disease progression. Baseline brain imaging was performed on a similar schedule. Among patients with T790M mutations, the objective response rate (ORR) was 62% (95% confidence interval [CI]: 54–68) in the AURA extension study (201 patients). In the AURA2 study (210 patients) the ORR was 70% (95% CI: 64–77) and the median PFS was 9.9 months (95% CI: 9.5–12.3) [21,22,23]. Docetaxel is the standard treatment for elderly patients based on the Japanese guidelines, as it provided an ORR of 22.7% in a study that compared docetaxel to vinorelbine [24]. Another recent study evaluated carboplatin plus pemetrexed for elderly Japanese patients and revealed an ORR of 41.2% [25]. Based on these findings, a required sample size of 31 patients was calculated according to the normal approximation method, with an expected response rate of 60%, a threshold response rate of 35%, two-sided alpha = 0.05, and 1 – beta = 0.8. However, the target sample size was increased to 35 patients to account for potential dropout cases. The primary endpoint for the trial was the overall response rate (ORR), while the secondary endpoints were PFS, OS, disease control rate (DCR), and safety events.

### 2.3. Statistical Methods

The ORR was calculated as the proportion of subjects with complete response or partial response as their best treatment responses. The DCR was calculated as the proportion of subjects who achieved stable disease (or better) as their best treatment response. The PFS interval was calculated from the date of enrollment to the first instance of disease progression, death from any cause, or the last follow-up without evidence of progression (for surviving patients with no evidence of progression). The OS interval was calculated from the date of enrollment to the date of death from any cause. Adverse events were evaluated from the first drug administration to 30 days after the last drug administration and were graded based on the Japanese JCOG translation of version 4.0 of the Common Terminology Criteria for Adverse Events.

The Wilson method was used to estimate the ORR and DCR with their two-sided 95% CIs. Statistical significance was considered present when the lower limit of the estimated 95% CI was above the threshold of 35% for ORR. The Kaplan–Meier method was used to evaluate the survival curves for PFS and OS, as well as the median and annual values. The Brookmeyer and Crowley method was used to estimate the CI values for median values, and Greenwood’s formula was used to estimate the standard error for annual values.

## 3. Results

### 3.1. Patient Characteristics

The study enrolled 36 patients between July 2016 and May 2018, with 23 female patients (63.9%) with a median age of 80 years, and 19 patients (52.8%) who were ≥80 years old. The histological types were adenocarcinoma in 35 patients (97.2%) and a mixed type with small cell lung cancer in only 1 patient. Based on the 7th edition of the AJCC system for staging lung cancer, 25 cases (69.4%) were considered stage IV, 10 cases (27.8%) involved relapse after surgery, and 1 case (2.8%) was considered stage IIIB. Among the enrolled patients, 30.6% were former smokers. The EGFR gene mutations involved the exon 20 T790M mutation in all cases, as well as exon 19 deletion in 22 cases (61.1%) and the exon 21 L858R point mutation in 11 cases (30.6%). Brain metastasis was detected in 15 patients (41.7%) (Table 1).

### 3.2. Efficacy

The ORR from our previous report was 58.3% (95% CI: 42.2–72.9), which included a complete response rate of 2.8% and a partial response rate of 55.6%. The stable disease rate was 38.9%, and the DCR was 97.2%. The median response duration was 54.9 weeks (95% CI: 26.9–69.1), and a waterfall plot revealed that 33 patients (91.6%) experienced tumor shrinkage, which indicated favorable antitumor activity. Sixteen patients (44.4%) continued treatment beyond progression.

**Figure 1:**
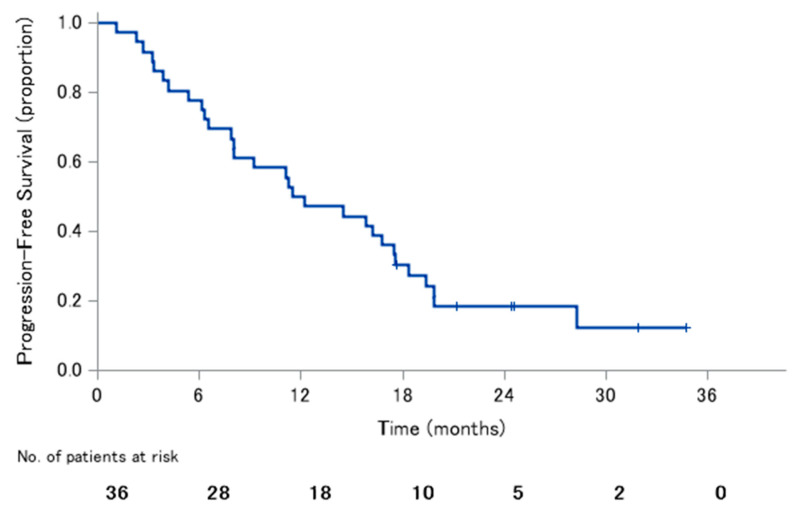
Progression-free survival.

**Figure 2:**
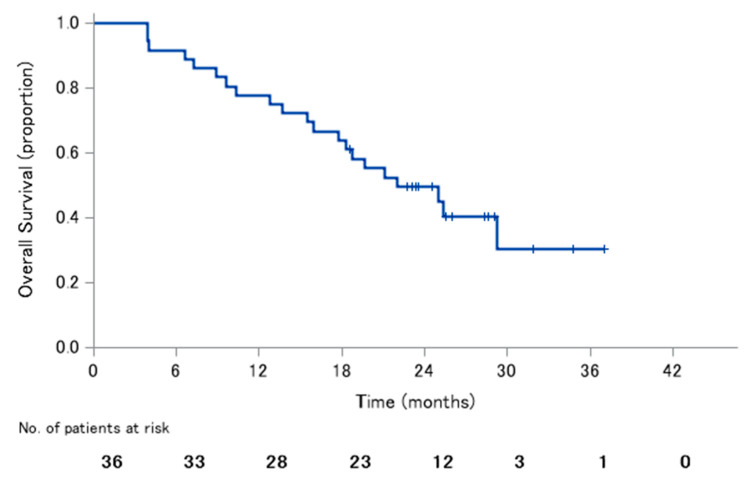
Overall survival.

The median PFS was 11.9 months (95% CI: 7.9–17.5), with 1-year PFS rate of 50.0% and 2-year PFS rate of 18.3% (Figure 1). The median OS was 22.0 months (95% CI: 16.0–not reached), with 1-year OS rate of 77.8% and 2-year OS rate of 49.5% (Figure 2).

### 3.3. Safety

Adverse events occurred in 31 cases (86.1%), with Grade 3 or higher adverse events observed in 10 cases (27.8%). Seven patients (19.4%) required dose reductions, 10 patients (27.8%) discontinued treatment because of adverse events, and 1 patient died (2.8%). The adverse event leading to death was a pulmonary infection, although this was judged unlikely to have been caused by the osimertinib treatment. There were no death events caused by drug-induced lung injury. The most frequent adverse event was anemia/hypoalbuminemia (27 patients, 75.0%), which was followed by thrombocytopenia (21 patients, 58.3%), paronychia/anorexia/diarrhea/neutropenia (15 patients, 41.7%), leukopenia/aspartate aminotransferase increase (14 patients, 38.9%), fatigue/acneiform eruption (13 patients, 36.1%), and alanine aminotransferase increase/alkaline phosphatase increase/creatinine increase (11 patients, 30.6%). The Grade 3–4 adverse events included fatigue, anorexia, diarrhea, cardiac ejection fraction decreased, prolonged QT, leukopenia, neutropenia, and aspartate aminotransferase increase. The cases of cardiac ejection fraction were decreased and the cases of prolonged QT were different cases, and delirium and hallucinations were observed in the same patient. Pneumonitis was observed in four patients (11.1%), including two patients (5.6%) with Grade 3–4 pneumonitis (Table 2).

## 4. Discussion

Treatment of NSCLC has advanced dramatically after the introduction of molecularly targeted drugs, such as EGFR-TKIs for EGFR-mutated cases. The first- and second-generation of EGFR-TKIs proved to be highly effective in several studies, although the effects tended to only last for approximately 1 year [1,2,3,4,5,6,7,8,9,10,11]. Approximately one-half of the resistant cases involved a gatekeeper mutation in exon 20 (T790M), and osimertinib was developed and approved for the treatment of these cases [12,13]. The results of the FLAURA trials positioned osimertinib as a standard treatment option, and even as an initial treatment option [14,15]. However, many cases still involve treatment in the second line or later, as the T790M mutation was identified via re-biopsy in patients who received first-generation or second-generation EGFR-TKIs as their initial treatment. When the T790M mutation was identified in these cases, patients typically received osimertinib.

Aging populations are becoming increasingly common worldwide, and many lung cancer cases involve older patients [26,27]. There are concerns that older patients have a higher risk of developing adverse events, which may necessitate dose reduction or treatment discontinuation, and subsequently result in decreased efficacy. Thus, this phase II study aimed to evaluate the safety and efficacy of osimertinib in elderly patients with EGFR-mutated lung cancer involving the T790M mutation. The primary endpoint was the ORR, and our previous report found that the ORR was 58.3% (95% CI: 42.2–72.9), which fulfilled the efficacy criterion (the lower limit of the CI exceeded the threshold response rate of 35%) [19]. This report describes the secondary endpoints, which include the DCR (97.2%), median PFS (11.9 months), and median OS (22.0 months). In terms of efficacy, the pooled results from the AURA expansion and AURA2 studies revealed an ORR of 66%, a DCR of 91%, a median PFS of 9.9 months, and a median OS of 26.8 months [23]. In addition, phase 3 AURA3 studies revealed an ORR of 70.6%, a DCR of 93.2%, a median PFS of 10.1 months, and a median OS of 26.8 months [13,28]. Thus, while our ORR was lower than that shown in the previous studies, it agrees with the slightly lower ORR (61.1%) that was retrospectively observed in another sample of elderly Japanese patients [29]. Furthermore, our findings regarding PFS and OS do not appear inferior to the results from previous studies, thereby suggesting that osimertinib was effective in elderly Japanese patients. Regarding the effects based on the PS, the ORR of PS0 and PS1 was 75% and 53.6%, respectively, and the PFS was 13.7 months and 11.9 months, respectively. Since there were few cases, it was impossible to discuss the significant differences, but the PS0 group tended to be superior.

It is also important to compare the results from osimertinib treatment to those from cytotoxic anticancer drugs, which are the alternative options if osimertinib is not used for T790M-positive cases. For example, the control group for the AURA3 study received platinum plus pemetrexed, which provided an ORR of 31%, a DCR of 74%, a median PFS of 4.4 months, and a median OS of 22.5 months [13,28]. A subgroup analysis of ≥70-year-old Japanese patients from the JACAL study evaluated carboplatin plus pemetrexed and revealed an ORR of 24%, a DCR of 68%, a median PFS of 5.2 months, and a median OS of 16.8 months [30,31]. Thus, our OS findings may be comparable to the results from the entire AURA3 population, although our ORR, DCR, and PFS outcomes are comparable or even slightly better. Interestingly, 71% of the patients in the group that received platinum plus pemetrexed subsequently received additional treatment, with 60% experiencing a greater effect after crossing over to osimertinib treatment. Therefore, while the JACAL study had only included EGFR-mutated cases and did not specifically consider older patients, we believe that osimertinib may provide good outcomes among older patients with EGFR-mutated (T790M) NSCLC.

Safety is also an important consideration in this setting, given the concerns regarding the potentially higher risk of adverse events among older patients. In the AURA3 study, it appears that Japanese patients had a higher risk of paronychia, diarrhea, and skin pruritus, although no clear increase was observed among elderly patients. However, elderly patients had a clearly increased frequency of myelosuppression events, such as anemia (75% in this study vs. 8% in AURA3 study), leukopenia (38.9% in this study vs. 8% in AURA3 study), neutropenia (41.7% in this study vs. 8% in AURA3 study), and thrombocytopenia (58.3% in this study vs. 10% in AURA3 study), although the frequencies of Grade 3–4 adverse events were generally comparable. Osimertinib has also been reported to be more frequently myelosuppressed than in other EGFR-TKI in a pivotal study [13,14]. In addition, myelosuppression was reported to be stronger in the analysis of the Japanese population [32]. Although the obvious mechanism was unclear, it was suggested that racial differences might be involved. Since myelosuppression was observed more frequently in the present study than in the aforementioned analysis of the Japanese population, caution should be exercised in the elderly Japanese. Fiala et al. reported that pre-treatment hypoalbuminemia correlated with poor prognosis in advanced NSCLC patients treated with erlotinib [33]. The present study also revealed that anorexia and exhaustion were common (30–40% of cases vs. 16-18% of cases in AURA3 study, including some Grade 3–4 cases), as well as hypoalbuminemia (75% of cases vs. N/A in AURA3 study). Therefore, careful follow-up is needed for elderly patients who are receiving osimertinib. Elevated alkaline phosphatase and creatinine values were also observed, albeit not serious cases, and related follow-up testing is also important. Cardiac adverse events, such as decreased left heart ejection fraction and QT prolongation, were observed in some cases, although only one patient experienced a Grade 3–4 cardiac adverse event. Central nervous system events, such as delirium and hallucination, may be explained by the large proportion of cases with brain metastasis (41.7%), although caution should be exercised if these events present in conjunction with sinusitis and pulmonary infection. Regarding AE by PS, no clear difference was observed between PS0 and PS1.

All-grade pneumonitis was observed in 11.1% of cases, and Grade 3–4 pneumonitis was observed in 5.6% of cases. The rates after conventional EGFR-TKI treatment were 4% in the AURA3 study and 7.3% in the Japanese subset of patients, which suggests that Japanese patients may have a higher rate of pneumonitis [13,34]. The difference between our findings and the previous findings may be related to differences in the proportions of patients with a history of smoking (69.4% for the present study, 32.2% for the AURA3 study, and 31.7% for the Japanese subset of the AURA3 population). In addition, the Japanese subset of the FLAURA study population had a higher frequency of pulmonary disorders (all grades: 12%, Grade 3 or higher: 2%); it should be noted that this is a first-line trial. Other reports have also suggested that osimertinib may be associated with an increased incidence of pulmonary disorders relative to other EGFR-TKIs [32]. Nevertheless, the odds ratio for pulmonary disorders after gefitinib treatment was 1.92-fold higher among Japanese patients who were ≥55 years old, which suggests that careful follow-up is required for patients who are ≥75 years old [35].

The present study revealed all-grade AEs in 86.1%, Grade 3 or worse AEs in 27.8%, and fatal AEs in 2.8% of the patients. These rates did not appear to be substantially elevated among elderly patients, based on results from the AURA3 study and its Japanese subgroup (all-grade: 97.8% and 100%, Grade 3 or higher: 22.6% and 31.7%, and fatal AEs: 1.4% and 0%). However, AEs leading to treatment discontinuation occurred in 12 patients (33.3%) in our study, which was more common than the rates of 6.8% in the AURA3 study and 7.3% in the Japanese subgroup. For example, we observed drug-induced lung injury in four patients (11.1%), and these patients needed to stop treatment. In addition, three patients (8.3%) discontinued treatment because of Grade 4 AEs (pulmonary infection, hallucinations, and hepatic dysfunction), although those events were judged unlikely to be associated with their treatment. One patient (2.8%) required a two-step dose reduction, and two patients (5.6%) were unable to continue the treatment protocol because of a ≥4-week treatment disruption. Treatment was also stopped in one case involving Grade 3 aspiration pneumonia, one case at the attending physician’s discretion, and one case because the patient refused to continue treatment. Thus, although the safety of osimertinib outside the study protocol has not been evaluated, most of these AEs and treatment discontinuations were likely not to have been caused by a drug-induced pulmonary injury.

Most all-grade adverse events involved anorexia, fatigue, myelosuppression, and gastrointestinal symptoms. These complications were generally not serious and could be addressed using conventional management strategies. However, it is important to note that the frequency of drug-induced lung injury may increase, which highlights the importance of a careful follow-up in this population. Despite the potential need for a careful follow-up and the small sample size, which was the limitation in this study, it appears that osimertinib can be a standard treatment even for the elderly patients harboring T790M mutation.

While the present study provided encouraging data, we are conducting an additional phase II study (SPIRAL-0) to confirm the safety and efficacy of osimertinib in ≥75-year-old patients with untreated NSCLC harboring EGFR-activating mutations [36]. This may provide further information to guide the increasing use of osimertinib treatment in this setting.

## Figures and Tables

**Table 1:** Patient characteristics.

		*n* (%)
Sex	Male	13 (36.1)
	Female	23 (63.9)
Age	Median (range)	80 (75–92)
	≥80 years	52.8 %
	>85 years	11.1 %
PS	0 1	8 (22.2) 28 (77.8)
Histology	Adenocarcinoma	35 (97.2)
	Adenocarcinoma + SCLC	1 (2.8)
Stage	IIIB	1 (2.8)
	IV	25 (69.4)
	Relapse after surgery	10 (27.8)
EGFR mutation	T790M	36 (100.0)
	Exon 19 deletion	22 (61.1)
	L858R	11 (30.6)
	G719X	1 (2.8)
Smoking status	Ex-smoker	11(30.6)
Pre-treatment	Surgery	12 (33.3)
	Chemotherapy EGFR-TKI Afatinib Erlotinib Gefitinib	13 (36.1) 36 (100.0) 5 (13.9) 10 (27.8) 21 (58.3)
	Radiotherapy	10 (27.8)
	Thoracic drainage	4 (11.1)
Metastasis site	Lung	18 (50.0)
	Pleural dissemination	12 (33.3)
	Brain	15 (41.7)
	Bone	12 (33.3)
	Liver	8 (22.2)

PS: performance status, SCLC: small cell lung cancer.

**Table 2:** Adverse events.

	Any Grade	Grade 3–4
All adverse events > 15%, *n* (%)		
Anemia	27 (75.0)	0 (0.0)
Hypoalbuminemia	27 (75.0)	0 (0.0)
Platelet count decreased	21 (58.3)	0 (0.0)
Neutrophil count decreased	15 (41.7)	1 (2.8)
Paronychia	15 (41.7)	0 (0.0)
Decreased appetite	15 (41.7)	4 (11.1)
Diarrhea	15 (41.7)	1 (2.8)
White blood cell decreased	14 (38.9)	1 (2.8)
Aspartate aminotransferase increased	14 (38.9)	2 (5.6)
Fatigue	13 (36.1)	3 (8.3)
Dermatitis acneiform	13 (36.1)	0 (0.0)
Alanine aminotransferase increased	11 (30.6)	0 (0.0)
Alkaline phosphatase increased	11 (30.6)	2 (5.6)
Creatinine increased	11 (30.6)	0 (0.0)
Pruritus	8 (22.2)	0 (0.0)
Mucositis oral	8 (22.2)	0 (0.0)
Grade 3–4 adverse events, *n* (%)		
Pneumonitis	4 (11.1)	2 (5.6)
Left ventricular systolic dysfunction	1 (2.8)	1 (2.8)
Electrocardiogram QT prolongation	1 (2.8)	1 (2.8)
Delirium	1 (2.8)	1 (2.8)
Hallucination	1 (2.8)	1 (2.8)
Dyspnea	1 (2.8)	1 (2.8)
Dehydration	1 (2.8)	1 (2.8)
Lung infection	2 (5.6)	1 (2.8)
Sinusitis	1 (2.8)	1 (2.8)
